# Assessing the vulnerability of freshwater fishes to climate change in Newfoundland and Labrador

**DOI:** 10.1371/journal.pone.0208182

**Published:** 2018-12-03

**Authors:** Hope O. Olusanya, M. van Zyll de Jong

**Affiliations:** 1 Environmental Policy Institute, Memorial University Grenfell Campus, Corner Brook, Newfoundland and Labrador; 2 Department of Biological Sciences, University of New Brunswick, Saint John, New Brunswick; Universidade de Aveiro, PORTUGAL

## Abstract

Freshwater fish populations are rapidly declining globally due to the impacts of rapid climate change and existing non-climatic anthropogenic stressors. In response to these drivers, freshwater fishes are responding by shifting their distribution range, altering the timing of migration and spawning and through demographic processes. By 2050, the mean daily air temperature is predicted to increase by 2 to 3 degrees C in insular Newfoundland and by 3 to 4 degrees C in Labrador. Mean daily precipitation is also projected to increase in all locations, with increased intensity projected for several regions. To mitigate negative consequences of these changes, managers require analytical approaches that describe the vulnerability of fish to climate change. To address this need, the current study adopts the National Marine Fisheries Service vulnerability assessment framework to characterize the vulnerability of freshwater fishes in Newfoundland and Labrador. Twelve vulnerability indicators were developed from an extensive literature review and applied to the assessment. Experts were solicited using an online questionnaire survey and scores for exposure, sensitivity and adaptive capacity were collated and analyzed to derive a final vulnerability score and rank for each species. The analysis showed one species to be of high—very high vulnerability, two species were highly vulnerable while four species were moderately vulnerable to climate change. The result provides insight into the factors that drive vulnerability of freshwater fishes in the region, this information is significant to decision-makers and other stakeholders engaged in managing freshwater fish resources in Newfoundland and Labrador.

## Introduction

The unprecedented rate and magnitude of climate change present a threat to global freshwater fish populations [1–3]. In temperate regions, the impacts to freshwater ecosystems will arise through elevated water temperatures, alterations in precipitation regimes, flow rates, onset and duration of ice cover, the frequency of disturbances such as wildfires, floods and insect infestations [4–7]. Since freshwater fishes are ectotherms, exposure to temperature-related stress can elevate physiological stress and increased metabolic demands, which directly affect growth, survival, reproduction and productivity [8–10]. In addition, temporal and spatial changes to precipitation will alter seasonal flow patterns having negative effects on critical life stages, phenology, population dynamics, and reproductive success [11–17]. Climate change can also alter the biotic components of freshwater ecosystems causing indirect impacts on freshwater fish populations. Impacts can be caused by increasing invasive species population size and distribution [16, 18, 19], changes in competition and predation rates [4] and, increases in disease risk and occurrence of parasites [20]. In some cases, freshwater fishes have responded to impacts through evolutionary change, spatial distributional shifts, alterations in demographic processes, or through changing the timing of seasonal migration and spawning [21]. The compound effect of climate change and other anthropogenic stressors have compromised the ability of fishes to cope with or respond to these changes at a pace that matches environmental changes [22]. Species are vulnerable when they possess only limited ability to adapt or respond to rapid rates of future climate change. These species face a greater risk of extinction or extirpation and are usually the focus of adaptation efforts [23–24].

The climatic projections for Newfoundland and Labrador based on the average output of seven regional climate changes models (RCM) [25] predicts a mean daily air temperature increase of 2 to 3 degrees Celsius in Newfoundland and 3 to 4 degrees Celsius in Labrador. Temperature shifts are predicted to be the greatest in winter and smaller in summer and autumn with increasing variations moving north toward Labrador. Mean daily precipitation is predicted to increase in all locations, with modest surges during the winter and spring with much smaller variation during summer [25]. Furthermore, increased intensity of precipitation is predicted for several regions with most events favouring rain over snow. To date, there have been no efforts to consider how these changes will affect freshwater fishes in the region. Studies conducted for other temperate regions in Canada have provided some useful insights on the impacts of warming on freshwater fishes in inland lakes and streams of boreal shield ecosystems [26]. For example, the elevated surface water temperature of shallow lakes [27], has been linked to a reduction in the availability of habitats and fragmented distributions of some cold-water fish species [26]. Elevated temperature has also been linked to increased stream flow, less ice cover during winter, drier summers with higher evaporation rates, and the potential for the transformation of suitable conditions to favour some cool and warm-water fishes [26, 28]. In summary, some fishes could experience net benefits and others net losses due to climate change. Despite lessons from such studies, the question remains if the projections for Newfoundland and Labrador will result in negative or positive impacts to its freshwater fish species. Coupled with this concern is the uncertainties that exist in climate models, and from a decision-making perspective, the need to plan in case future events mirror current predictions. Understanding and evaluating vulnerability can be useful in this situation to inform adaptation management actions while acknowledging uncertainty [29]. Therefore, there is the need to profile the vulnerability of freshwater fishes in Newfoundland and Labrador to climate change.

Vulnerability assessment is a key activity in developing adaptation options for conservation outcomes [29]. The outcomes from a vulnerability assessment process are the identification and triaging of the vulnerable components within a system [30]. This presents a platform for the decision-maker to engage in further analysis or to justify strategic management actions. In this study, we aim to assess the vulnerability of freshwater fishes in Newfoundland and Labrador to climate change. The study uses a trait-based method in concert with expert knowledge. The objective of this study is to adapt the National Marine Fisheries Service (NMFS) [31] vulnerability assessment framework to assess the vulnerability of freshwater species in Newfoundland and Labrador, Canada.

## Methods

The National Marine Fisheries Service (NMFS) climate-change vulnerability framework [31] was adapted for the purposes of this study. The modified NMFS framework possessed three phases: a scoping phase, an assessment phase and an analysis phase. The scoping phase comprised of activities that shaped the boundary and objectives of the research. The steps in this phase included: determining the spatial and temporal scale of the assessment, selecting the target species, identifying relevant climatic factors, selecting appropriate sensitivity and adaptive capacity indicators relevant to the study objective and finally, identifying and selecting appropriate individuals with expertise in the subject area. The assessment phase represents the actual process of evaluating the vulnerability of the species. It involved the following activities: designing the online questionnaire survey and conducting the expert elicitation process. During the analysis phase, data are collated, analyzed and visualized; this includes collating experts scores, conducting a quantitative data analysis of the scores, and determining the overall vulnerability of the species.

### Study area and species

Newfoundland and Labrador, features various freshwater lakes, natural and regulated rivers, pristine wetlands and boreal forests providing habitat to anadromous and landlocked freshwater fish populations [32]. The freshwater fish fauna of the region is composed of 15 native species in insular Newfoundland with an additional 12 in Labrador for a total of 27 native species [33, 34]. In addition, 5 non-native salmonid species were introduced in the 1880s in response to fishery policies advocating the stocking of exotic species [33, 34]. Information on the distribution and life history variation of freshwater species in the province is limited. For the purposes of this study, vulnerability assessment was confined to those species that have well-documented information on species and population distribution, habitat requirements and life history variation combined with available scientific expertise. The target species that met these criteria include; Atlantic Salmon (*Salmo sala*, Linnaeus, 1758), Brook Trout (*Salvelinus fontinalis*, Mitchill, 1814), Lake Trout (*Salvelinus namaycush*, Walbaum, 1792), Brown Trout (*Salmo trutta*, Linnaeus, 1758), Arctic Char (*Salvelinus alpinus*, Linnaeus, 1758), Northern Pike (*Esox Lucius*, Linnaeus, 1758) and Rainbow Trout (Oncorhynchus mykiss, Walbaum, 1792).

### Vulnerability indicators

Based on the IPCC’s definition [35], the climate change vulnerability of a species is characterized by three factors: exposure, sensitivity and adaptive capacity. In this study, exposure refers to the magnitude of climate change factors (temperature and precipitation) projected to impact freshwater habitats in the study area [21, 31]. Projected changes in air temperature served as a proxy for changes in water temperature while projected changes in precipitation served as a proxy for stream flow since these factors correlate in freshwater ecosystems [31]. Sensitivity to climate change refers to the degree of persistence displayed by a species in the presence of changing climate factors [36]. It reflects the intrinsic ability to withstand changes in the environment; therefore, sensitive species will possess limited survivability to climate change [37]. Adaptive capacity points to the inherent ability to adapt or move to suitable habitats and is potentially determined by behavioural changes, dispersal ability, and genetic variation [37]. Sensitivity and adaptive capacity are based on freshwater fish’s intrinsic traits [30]. A review of the published literature identified relevant life history and biological factors [21, 38, 39, 40], nine of which formed the vulnerability indicators. S1 Table provides a list of these indicators and its definition. Anthropogenic stressors were included as an element of the vulnerability indicators. Overall, 12 indicators were developed and used in this assessment.

### Expert selection

Twenty-six individuals were identified as our potential experts for the assessment. The individuals consisted of freshwater fisheries scientists from government research agencies, conservation groups, and academia. Participants in the survey were chosen based on whether they had extensive research experience with freshwater fishes in the study area and that they possessed PhD or it’s equivalent. All experts were assumed to have adequate technical knowledge of all selected species including their distribution, life history, population dynamics and status and major drivers of change and current and planned management regimes. It was also assumed that all experts could obtain new and existing information on all species with which to make sound judgments to assess the species. Lastly, experts were deemed to have the ability to articulate the justification for their assessments.

### Data collection and analysis

An online questionnaire was developed and employed to facilitate data collection. Participants accessed the online questionnaire through Survey monkey (an online survey tool). The questionnaire was designed into five sections consisting of twelve survey questions each relating to a vulnerability indicator. In the first section of the survey, experts assessed the exposure to projected changes in water temperature and precipitation (stream flow change) for each freshwater fish. The expert assessment was aided by climate change projections provided in [25] and [41]. In addition, the Nature Conservancy’s climate wizard tool was provided as a link in the questionnaire to supplement expert knowledge. This tool provides spatially explicit visual maps of the study area using an ensemble of statistically downscaled Global circulation models (to a 0.5-degree grid) to project average annual temperature and precipitation changes by mid-21st century [42]. In the second and third sections, experts assessed sensitivity and adaptive capacity indicators respectively for all species. The fourth section assessed the cumulative effects of non-climatic stressors for each species distribution range. The final section required participants to indicate other threats or species-specific vulnerability factors not included in the survey.

Experts were required to provide three types of ordinate scores: an indicator score (for exposure, sensitivity, adaptive capacity and cumulative non-climatic factors), weight scores (to determine the relative importance of an indicator to the overall species vulnerability) and confidence scores (to account for uncertainty in the scoring). A scoring scale of low, moderate, high and very high was used for all vulnerability indicators while low, moderate and high, was used for weight scores and confidence scores. Numerical values of 1 to 3 were attributed to the scoring scales of the weight and confidence scores where low = 1, medium = 2, high = 3. In assigning numerical values to vulnerability indicators, the scoring bins for exposure and sensitivity were attributed values corresponding to low = 1, moderate = 2. high = 3 and very high = 4. In this way, the higher the exposure and sensitivity index for a species the higher its overall climate change vulnerability. Since adaptive capacity has an inverse relationship with vulnerability [37], the numerical values attributed to the scoring scale was reversed. Therefore, adaptive capacity had values corresponding to low = 4, moderate = 3, high = 2, very high = 1. Using this, the higher the adaptive capacity index (i.e. low vulnerability) the higher the climate change vulnerability. Each indicator score was calculated using the weighted means of the expert’s scores. The exposure, sensitivity and adaptive capacity index scores were derived using a modified logic rule [31] instead of averaging the respective indicator scores. Simply averaging all indicator scores has been noted to de-emphasize the effects of high scoring indicators on a species vulnerability [21]. Hence, to account for the significance of high scoring indicators, a logic rule was applied, resulting in an adjusted exposure, sensitivity and adaptive capacity index. The logic rule was applied when more than two indicator-scores within the exposure, sensitivity and adaptive capacity component was greater than 3.0, therefore, the final component score was adjusted by 0.5. The logic rule ensures that the final vulnerability score of the species reflects the potential for ‘multiple risks and highlights species with ‘life history requirements that environmental changes could impact through multiple mechanisms’ [21]. The final cumulative vulnerability score for each species was computed by adding the adjusted scores for exposure, sensitivity and adaptive capacity index: V = f (E, S, AC), where, V = cumulative vulnerability score, E = exposure index, S = sensitivity index and AC = adaptive capacity index. The final scores, therefore, ranged from 3 (minimum) to 12 (maximum). Species with higher scores were placed in a higher vulnerability rank to the projected changes by mid-century. Seven rank levels were developed to categorize the vulnerability score. No specific standard currently exists in literature to set vulnerability thresholds [43], hence, to present an easy mechanism to communicate and rank vulnerability, the categories were evenly sub-divided (Table 1). Uncertainty was analyzed by averaging expert’s confidence scores for each indicator. A scatterplot was developed to highlight the potential climate impact on the species. A Mann-Whitney U rank test was conducted on the expert’s vulnerability scores to determine if there were significant differences between native and non-native species. Finally, to ascertain the factors driving vulnerability of freshwater fish to climate change, the average of all vulnerability indicators across all species was computed. All computations were derived using Microsoft office Excel 2013 and Minitab Statistical Software.

**Table 1:** Climate change vulnerability scores and rank categories applied across the freshwater fishes.

Vulnerability score	Rank Category	
3.0–5.0	Low	
5.1–5.9	Low–moderate	
6.0–8.0	Moderate	
8.1–8.9	Moderate-High	
9.0–10.0	High	
10.1–10.9	High—very high	
11.0–12.0	Very high	

### Ethical considerations

The study relied on expert opinion and therefore was subject to the human ethical review process of the Grenfell Campus Research Ethics Board (GC-REB). The board is governed by the Ethics of Research Involving Human Participants policy and follows the Tri-Council Policy Statement on Ethical Conduct for Research Involving Humans 2 (TCPS2). Potential participants were provided with a recruitment letter and written informed consent describing the purpose of the study, the expectations from the participants in the study, duration, possible benefits and risks, duration, and how confidentiality and anonymity would be maintained prior to the commencement of the survey. The GC-REB approved the study to be in ethical compliance with the Tri-Council policy on ethics in human research (TCPS2).

## Results

Eleven experts (42%) from a limited pool of 26 experts answered the assessment survey. Of those returned surveys 73% (n = 8) had all sections completed. Two rounds of email reminders were sent to participants who had not responded to the survey i.e. corresponding to 58% of the total participants and those with incomplete surveys to increase the response rates. Bearing little result, the research team felt that the final number of respondents represented an acceptable proportion of the identified pool of experts and thus decisively used only completed surveys for this analysis. In total this corresponded to 31% of identified experts. The justification for this decision was that similar surveys with limited pools of expertise had been used to make sound and useful judgement to conservation planning [44, 45]. All participants in this study were selected from a limited available pool of experts in freshwater fish ecology and management in the province of Newfoundland and Labrador. Also, considering the geographic scope and a limited number of species the response rate is acceptable for this study.

**Fig 1:**
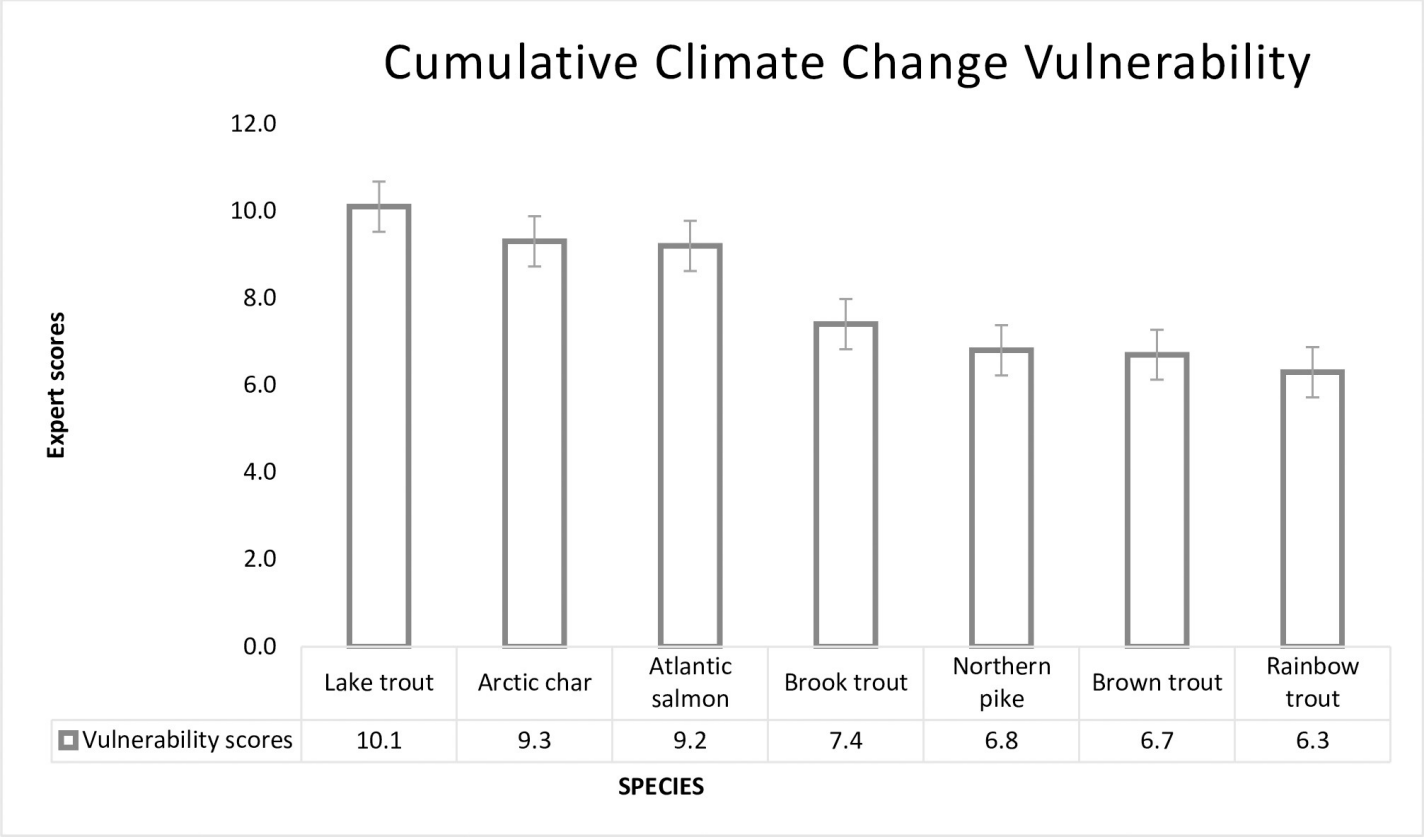
Relative climate change vulnerability scores of selected fishes. The selected fish species are ranked per their relative vulnerability, based on freshwater fish expert’s scoring (n = 8).

Exposure index scores ranged from moderate to very high indicating the magnitude of projected changes in water temperature, precipitation and anthropogenic impacts to the fish’s distribution and spatial range. The lowest exposure was predicted for Northern Pike while the highest exposure was predicted for Atlantic salmon. The sensitivity index for the species showed moderate or high sensitivity to projected changes in climatic and non-climatic factors. Atlantic Salmon and Arctic Char showed the highest sensitivity to these changes. Adaptive capacity ranged from low to moderate. None of the target species were predicted to exhibit high adaptability to projected changes. Lake Trout and Northern Pike were rated low in their adaptability to future changes. In terms of the overall climate change vulnerability, four species were ranked moderately vulnerable, two species highly vulnerable and one species high—very highly vulnerable (at a transition point) to future changes (Fig 1). No species was ranked with a low vulnerability (Table 2). Comparing the expert's scores among native and non-native species showed that native species have a relatively higher vulnerability to climate change than non-native species. This points to the possibility that by mid-century, climatic conditions may favour non-native or other exotic species than native or endemic species. A Mann-Whitney u rank sum test of the expert scores showed a statistically significant difference between native and non- native fishes (p = 0.003) suggesting experts expect higher vulnerability for native species compared to non-native species in the region.

**Table 2:** Climate change vulnerability scores of selected fishes, from regional fish experts (n = 8). Species are ranked from highest to lowest vulnerability. * indicates non-native species. Exposure, sensitivity and adaptive capacity were calculated using the weighted means of all indicators and applying a logic rule to derive a cumulative vulnerability for all species.

Species	Exposure	Sensitivity	Adaptive Capacity	Vulnerability	Vulnerability rank
**Lake Trout**	3.4	3.1	3.6	10.1	High—very high
**Arctic Char**	3.1	3.2	3.0	9.3	High
**Atlantic Salmon**	3.6	3.2	2.4	9.2	High
**Brook Trout**	2.5	2.3	2.6	7.4	Moderate
Brown Trout*	2.3	2.0	2.4	6.7	Moderate
Northern Pike*	1.5	1.7	3.6	6.8	Moderate
Rainbow Trout*	2.0	1.9	2.4	6.3	Moderate

**Fig 2:**
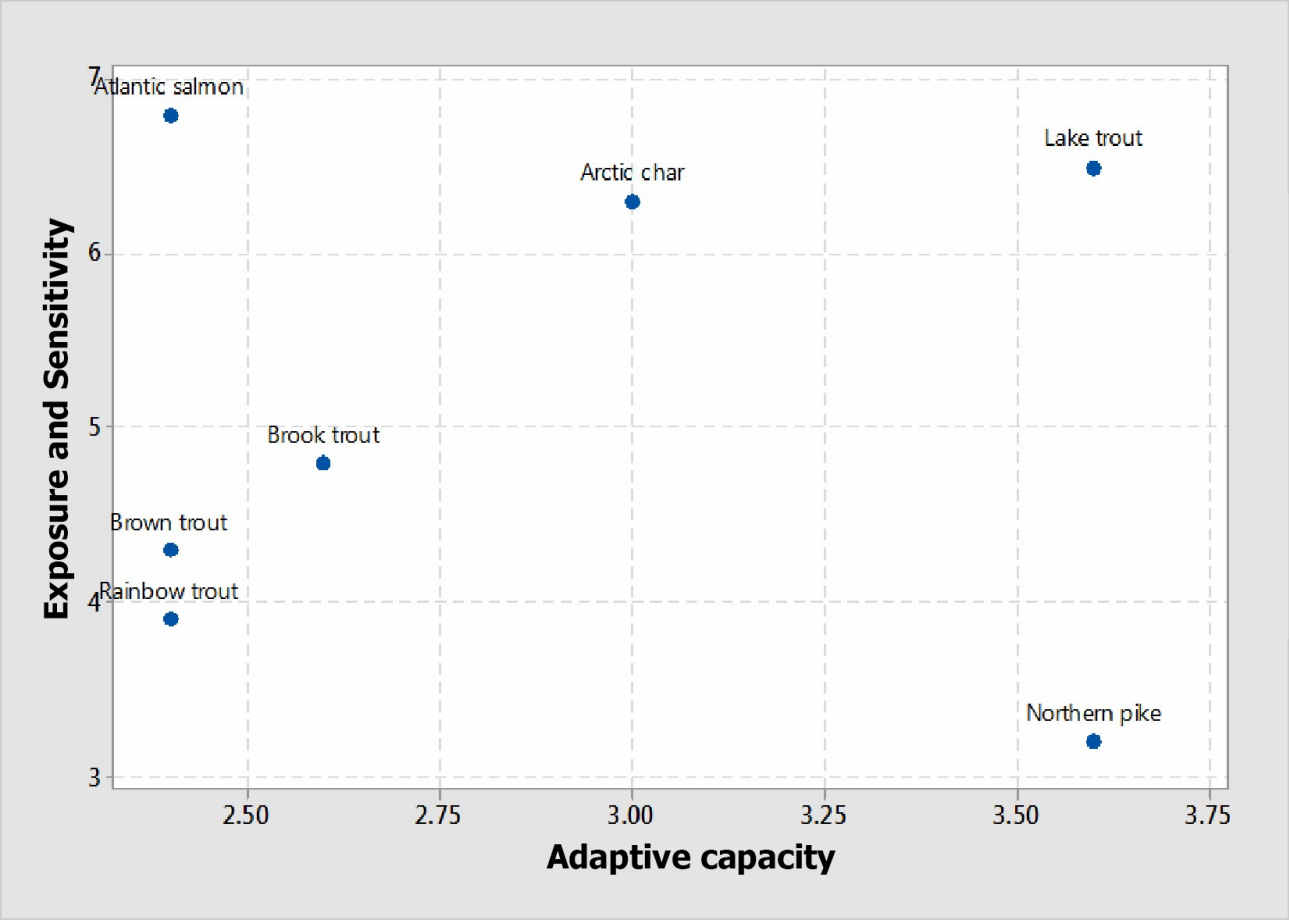
Scatterplot showing the relationship between exposure/sensitivity and adaptive capacity for the selected fishes.

The scatterplot (Fig 2) demonstrates the potential climate impact for each species within each quadrant. A species with high exposure, high sensitivity and low adaptive capacity to withstand changes fall within the high climate impact/vulnerability. Species likely to experience higher climate impacts are located on the upper quadrant and vice versa while species occupying the right side displays limited adaptability and vice versa. The most vulnerable species can be viewed at the uppermost right quadrant and vice versa.

Table 3 highlights the analysis of mean expert’s confidence scores across the selected species. From this, it is observed that on average, expert’s confidence ratings were moderate for exposure, sensitivity, adaptive capacity and vulnerability across all species. The individual expert’s confidence scores across all indicators were analyzed to ascertain if there was agreement in these ratings across all species. An analysis of variance (ANOVA) was computed and the result showed no statistically significant difference among expert’s confidence ratings towards all the selected species (p = 0.75).

**Table 3:** Average expert confidence scores (n = 8). A scale of 1–3 (where 1 = low confidence, 2 = medium confidence and 3 = high confidence) was used to represent expert confidence. The confidence ratings across all indicators were averaged to derive the final confidence scores, for all vulnerability components (exposure, sensitivity, and adaptive capacity). * indicates non-native species.

Species	Exposure	Sensitivity	Adaptive capacity	Vulnerability
**Lake Trout**	2.2	2.3	2.3	2.3
**Arctic Char**	2.3	2.3	2.4	2.4
**Atlantic Salmon**	2.5	2.4	2.5	2.4
**Brook Trout**	2.3	2.3	2.3	2.3
Brown Trout*	2.0	2.2	2.3	2.3
Northern Pike*	2.0	1.9	2.0	2.0
Rainbow Trout*	2.1	2.2	2.3	2.3

### Drivers of climate change vulnerability

To ascertain which factors were likely to contribute the most to the final climate change vulnerability in the region, the mean experts score for all indicators across the species was calculated. Indicators having higher scores represent those factors that would strongly influence climate change vulnerability in the region. Table A in S2 Appendix shows the extent to which all indicators influence the overall vulnerability while Figure A in S2 Appendix provides a comparison across all indicators. From the analysis, it is observed that inherent resilience, dispersive capability, dependence on environment cues impacted by climate change, exposure to temperatures and genetic plasticity constitute the major drivers of freshwater fish species vulnerability in the region. On the other hand, it can be observed that species vulnerability is driven by different contributing factors. For instance, Figure B in S2 Appendix indicates that Lake Trout’s relative vulnerability (high–very high), is majorly influenced by a combination of 7 factors, while Rainbow Trout’s relative vulnerability (moderate) is mostly influenced by 6 factors. Figure B in S2 Appendix provides further information for all assessed species.

To identify the non-climatic factors that will interact with climatic changes to drive species vulnerability, the section of the survey which asked experts to indicate the likely non-climatic threats to each species was analyzed and categorized into 6 sub-headings (Table 4). In total, 128 responses were generated by all experts for all the species assessed. These responses suggest that habitat degradation/loss is the most threatening non-climatic factor to a freshwater fish vulnerability in Newfoundland and Labrador. For Lake Trout and Northern Pike, 50% of expert responses i.e. 7 out of 14 responses for both species were related to habitat loss and degradation. Over-exploitation was another critically important non-climatic threat indicated by the experts’ responses.

**Table 4:** Non-climatic drivers of vulnerability for the selected species. Experts provided a total of 128 responses which were grouped into 6 categories. * indicates non-native species.

Species	Habitat loss	Invasiveness/Predation/ competition	Sea mortality	Overexploitation	Disease and pollution	Aquaculture
Lake Trout	7	1	0	5	1	0
Arctic Char	6	1	1	5	1	1
Atlantic Salmon	8	4	2	6	2	2
Brook Trout	8	4	1	6	2	0
Brown Trout*	9	3	2	5	2	1
Northern Pike*	7	1	0	5	1	0
Rainbow Trout*	5	2	1	4	2	1

## Discussion

Globally, climate change stressors are increasing the vulnerability of freshwater fishes. Native species are at the higher end of this vulnerability due to their dependence on cold, clean water [28]. There is evidence that several species in Canada are already experiencing the negative impacts of climate change which would likely continue in the future [4]. Recent reports from the Committee on the Status of Endangered Wildlife in Canada (COSEWIC) also confirm that the impacts of multi-faceted threats (including climate change) are driving the declining populations of some freshwater fishes [46]. Hence resource managers need to capitalize on available data such as the climate change projections for Newfoundland and Labrador to provide place-based adaptive management responses.

From the analyses presented in this paper, climate change vulnerability ranked from moderate to high—very high vulnerability; no species was ranked low in climate change vulnerability. S1 Appendix discusses the driving factors of each species vulnerability. Considering that this assessment did not include all freshwater fish species recorded in the province, it is indeterminate if similar results will occur on a broad scale assessment for the region. Notwithstanding, this result is in line with findings from studies conducted in other temperate latitude regions which suggest that native species are more vulnerable to climate change and non-native species especially warm or cool water fish species may likely expand their northward range [4, 47].

Climate change may outpace the ability of some species to shift to suitable habitats and genetically evolve hence the need for proactive management responses cannot be overemphasized. Conservation management in Newfoundland and Labrador would be improved by reinforcing existing practices with adaptation management approaches [48]. Adaptation planning and implementation frameworks links known climate change vulnerability drivers with specific conservation management objectives [49]. For instance, species predicted to be highly sensitive to temperature changes, could benefit from management actions implemented at restoring riparian forests [26], thus enhancing the resilience of the target species to climate change. In instances where species have limited dispersal ability to migrate or colonize suitable habitats or in situations where such an opportunity has been truncated by anthropogenic activities, management efforts could aim at addressing these challenges [26]. Adaptation strategy could focus on enhancing the natural evolutionary responses or adaptive capacity of vulnerable species to environmental changes through conservation activities like assisted migration, translocation or enhancing habitat connectivity.

## Conclusion

The NMFS methodology presented a step by step useful guideline for the assessment. Expert’s knowledge synthesized through systematic indicator scoring facilitated through an online questionnaire survey presented a unique mechanism to characterize the vulnerabilities of seven freshwater fish. Analysis of the results supported claims that native species were relatively more vulnerable to climate change. Vulnerability ranks ranged from moderate to very high vulnerability. Several limitations were recognized in this study. This assessment could represent a first step effort to quantify the climate change vulnerability of freshwater fish in the province. Future assessments could produce more refined results by employing spatially explicit vulnerability assessments with species traits [50]. Ultimately, the quantified results presented by this study represents a relative measure of climate change vulnerability since the results lack the capacity to serve as an absolute measure of vulnerability. The scope of this assessment was limited to the impacts of temperature and precipitation as the climate factors. Future assessments should consider expanding this to include multiple climatic variables relevant to freshwater ecosystems as more robust climate models for the region emerges. In addition, out of the 27 freshwater fishes known to occur in the region, this assessment covered 7 species due to the availability of required data and expertise. Data synthesis on other species should be complete to allow for further assessments on all freshwater fish species not included in this study. However, from a planning and decision-making perspective, this study could be significant for conservation, to inform climate change adaptation planning, prioritizing monitoring and further research for freshwater fish. Since climate change would likely outpace the ability of some species to shift to suitable habitats and genetically evolve, the need for proactive management responses cannot be overemphasized. Adaptation management actions such as assisted translocation or migration, removal of non-climatic anthropogenic stressors, habitat connectivity restoration, and restoring riparian forests are some responses that can strategically target vulnerable species and habitats.

## Supporting information

S1 Table Definition of vulnerability traits indicators. (DOCX)

S1 File Data sheet showing expert opinion scores and analyses. (XLSX)

S1 Appendix Species narratives. (DOCX)

S2 Appendix Drivers of climate change vulnerability. (DOCX)
