# Supplementary material for: Assessing the vulnerability of freshwater fishes to climate change in Newfoundland and Labrador
Source: PLoS One. 2018 Dec 3;13(12):e0208182. doi: 10.1371/journal.pone.0208182 (PMC6277096; doi:10.1371/journal.pone.0208182)
Supplement: S1 Table — (DOCX) [file pone.0208182.s001.docx]

**S1 Table: Definitions of vulnerability indicators used in the study**

| **Indicators** | **Definition** | **References** |
| --- | --- | --- |
| **Habitat specialization** | This indicator defines the relative dependence of a species on its ability to thrive in a wide range of habitat or its restriction to specific habitats throughout its range. Species could be considered as habitat specialists or generalists. This trait provides an understanding about a species ability find and inhabit suitable habitats if forced out of its current distribution as a result of possible stochastic events from climate or non-climate changes. | Morrison et al 2015,  Pacifici et al 2015,  Foden 2015,  Foden et al 2013,  Moyle et al 2013,  Galbraith and Price 2009 |
| **Prey specificity/diet**  **choices** | This indicator possibly determines indicates the relative flexibility of a fish species feeding habits. Species could be considered prey generalist or a prey specialist. | Morrison et al 2015,  Pacifici et al 2015,  Foden 2015,  Foden et al 2013,  Moyle et al 2013,  Galbraith and Price 2009 |
| **Physiological/behavioral**  **sensitivity to temperature changes** | This indicator points to the physiological and behavioral tolerance of a species at various life stage to exposure temperature considering the anticipated temperatures projections. | Chin et al 2010  Stortini et al 2015  Morrison et al 2015,  Pacifici et al 2015,  Foden 2015,  Foden et al 2013,  Moyle et al 2013,  Galbraith and Price 2009 |
| **Physiological/behavioral**  **sensitivity to changes in precipitation or river**  **flow** | This indicator points to the physiological and behavioral tolerance of a species (at various life stage) to projected changes in precipitation events and flow regimes. | Chin et al 2010  Stortini et al 2015  Morrison et al 2015,  Pacifici et al 2015,  Foden 2015,  Foden et al 2013,  Moyle et al 2013,  Galbraith and Price 2009 |

| **Population**  **size/Geographic range** | This indicator assesses a species population health, and therefore its ability to cope with negative climate change impacts. Species with known declining status, population trend or distribution range could exhibit lower resilience to climate change than species with a growing population or distribution. | Morrison et al 2015,  Pacifici et al 2015,  Foden 2015,  Foden et al 2013,  Moyle et al 2013,  Galbraith and Price 2009 |
| --- | --- | --- |
| **Dependence on**  **environmental cues likely to be disrupted by climate change** | This factor assesses the dependence of a species throughout its life stages on specific environmental triggers or cues e.g. migration, spawning of freshwater fish may be altered in their timing and magnitude by projected climate change. Species having strict dependence on environmental cues may be more sensitive to climate change impacts. | Morrison et al 2015,  Pacifici et al 2015,  Foden 2015,  Foden et al 2013,  Moyle et al 2013,  Galbraith and Price 2009 |
| **Genetic plasticity and**  **evolvability** | This factor relates to the ability of fish population genetic ability to rapidly evolve at the same pace with projected climate change. Fish populations with relatively high genetic variation and capacity to evolve will likely be more adaptable to changes in climate than species with lower genetic variation. | Morrison et al 2015,  Pacifici et al 2015,  Foden 2015,  Foden et al 2013,  Moyle et al 2013,  Galbraith and Price 2009 |

| **Dispersive capability** | This indicator is used to assess the relative dispersive capabilities of the species. This trait could be important for instance to determine the ability of adult species to migrate beyond natural and anthropogenic barriers or through larger distribution of eggs the greater probability to adapt to a shifting climate envelop thereby colonizing new habitats. Species with poor dispersive capability would be limited in this ability. | Schloss et al 2015  Barber et al 2016  Morrison et al 2015,  Pacifici et al 2015,  Foden 2015,  Foden et al 2013,  Moyle et al 2013,  Galbraith and Price 2009 |
| --- | --- | --- |
| **Inherent resilience** | This factor assesses the innate resilience of a species using their generation time, size and age at maturity, and fecundity or productivity. This trait is a determinant of a species adaptability. For example, species with relatively higher fecundity and generation time could likely adapt to climate change. Long-lived species that reproduce infrequently could have limited adaptability. | Morrison et al 2015,  Pacifici et al 2015,  Foden 2015,  Foden et al 2013,  Moyle et al 2013,  Galbraith and Price 2009 |
