# Supplementary material for: Assessing the vulnerability of freshwater fishes to climate change in Newfoundland and Labrador
Source: PLoS One. 2018 Dec 3;13(12):e0208182. doi: 10.1371/journal.pone.0208182 (PMC6277096; doi:10.1371/journal.pone.0208182)
Supplement: S1 Appendix — (DOCX) [file pone.0208182.s003.docx]

**S1 Appendix: Species narrative**

**Lake Trout *(Salvelinus fontinalis)* - high - very high vulnerability**

Lake Trout populations are distributed mostly in Southern Labrador, no population is recorded to be established in insular Newfoundland [1, 2]. Experts rated Lake Trout to be high to very high (high – very high) vulnerable to future climate change. Expert’s scores showed that exposure to changing stream and lake temperatures and high physiological and behavioural sensitivity to temperature are among the major risk factors for the species. Previous studies document that adult and juvenile have strong temperature and oxygen preferences and occupy shallow lakes and rivers of approximately 10oC [1, 2] and deep portions when the temperature exceeds 15oC [3]. Climate impacts studies focused on boreal shield lakes show that future warming could impose increased thermal stress on Lake Trout populations [4]. Experts assessed that biological factors such as habitat specialization, relatively low dispersal ability, resilience and genetic plasticity could increase this risk of vulnerability for Lake Trout in the region. More than 80% of non- climatic threats to Lake Trout species were categorized under habitat degradation and over-exploitation (i.e., illegal and legal). Such threats include: instream barriers, toxins, and invasive species, also posed a current and future vulnerability risk to Lake Trout population.

**Atlantic Salmon (*Salmo salar*) - high vulnerability**

Atlantic Salmon was ranked highly vulnerable to climate change and expert’s confidence scores were the highest among other species. Atlantic Salmon is reported throughout Newfoundland and southern Labrador existing as anadromous and landlocked populations and is observed to prefer clean, cool waters with specific micro-climates [1, 2]. Expert’s high vulnerability rank for Atlantic Salmon results from a combination of increased exposure to temperature changes, high physiological/ behavioural sensitivity to temperature, sensitivity to precipitation and its high dependence on environment cues likely to be interrupted by climate change. This shows some parallels with existing literature and assessments. For instance, Atlantic Salmon has been shown to have a maximum critical temperature threshold of about 20oC (depending on the life stage) [5]. Atlantic Salmon’s relatively high score for habitat specialization shows some limitations to its ability to colonize new habitats. About 75% of anthropogenic stressors listed by experts threatening Atlantic Salmon related to habitat loss, invasive species and overexploitation of the species. Similar factors were observed in other vulnerability assessments studies [6, 7].

**Brook trout *(Salvelinus fontinalis) -* moderate vulnerability**

Brook Trout is a native species distributed across Newfoundland and Labrador [8]. Experts rated the species moderately vulnerable to climate change due to factors such as moderate exposure to increasing temperature and precipitation. Brook Trout are documented to prefer cool, well-oxygenated headway streams, rivers and gravely lakes [1, 2]. Significant mortality occurs when water temperature exceeds 25oC [9] hence sensitive to temperature as observed in expert’s scores. Low dispersal ability and low inherent resilience presented by expert’s scores suggest some biological limits to Brook Trout adaptability. These factors together with non-climatic stressors such as industrial development, invasiveness, competition with other salmonids, were noted as threats to Brook Trout’s population.

**Brown Trout *(Salmo trutta)-* moderate vulnerability**

Brown Trout a non-native species known to exhibit similar environmental requirements with Brook Trout [2, 8] was ranked moderately vulnerable with expert scores showing moderate confidence ratings. Expert ratings suggested a relatively higher physiological and behavioural tolerance to temperatures. This could indicate that future conditions may present Brown Trout with a northward range expansion possibly out-competing fishes like Brook Trout or other salmonids that show higher sensitivity to temperature changes [10]. However, biological factors such as low genetic plasticity, low inherent resilience and dispersive capacity were considered to increase its climate change vulnerability. In addition, threats from instream barriers, dams, habitat modification/fragmentation, constitute some of the non-climate factors noted by experts to enhance vulnerability risk of Brown Trout.

**Rainbow Trout *(Oncorhynchus mykiss)* - moderate vulnerability**

Rainbow Trout also a non-native species of Newfoundland described [1, 11] is well adapted to clear cold deep lakes and have also been reported in smaller lakes, ponds and streams environments [2]. Ranked as moderately vulnerable to climate change, experts assessed that low adaptability in terms of its inherent resilience and dispersal capability compared to its exposure and sensitivity to future changes were determinants of its vulnerability hence it showed the least climate change vulnerability. Anthropogenic stressors like habitat loss and harvest (legal and illegal) could drive climate vulnerability for Rainbow Trout. Actions to limit anthropogenic pressures on habitats (such as habitat protection measures) could be sufficient for conserving this species [12] through enhanced resilience and dispersal capability to shift its distribution in response to climate suitability.

**Arctic Char *(Oncorhynchus mykiss) -* high vulnerability**

Arctic Char’s distribution extends from some parts of Newfoundland to the northern coasts of Labrador [1, 2]. Arctic Char was considered highly vulnerable to future climate change from this assessment. Exposure to rising water temperature was considered to highly increase its vulnerability. Factors such as high physiological tolerance to temperature, high habitat specialization and dependence on environmental cues were, in addition, important determinants of high sensitivity to future changes. Combining limited adaptability traits (low genetic plasticity, resilience) were also noted to likely enhance future climate change vulnerability.

**Northern pike *(Esox lucius****)-* **moderate vulnerability**

Northern pike is distributed mostly throughout southern Labrador [1, 2] and was rated moderately vulnerable to projected climate change. Trait factors such as high physiological/ behavioural tolerance to temperature and precipitation suggest that direct thermal stress from exposure to temperature would unlikely occur [13] reflecting some potential to expand its range. Scientific studies have observed the species to exhibit a high tolerance to a wide range of environmental conditions with an upper lethal temperature limit of 29oC [14]. Low habitat specialization and a notable prey generalist (Beaudoin et al., 1999) the species possess some potential to persist and colonize habitats under changing conditions. Conversely, [13] suggested that changing prey abundance (usually other salmonids like Arctic Char) due to climate change could increase Northern pike’s risk of climate change vulnerability. Also, the analysis showed that low relative adaptive capacity factors such as limited dispersal capability for example during adult stages (except during migration and spawning) are known to be mostly sedentary [14], and its early life stages are dependent on environmental cues which could increase the risk of vulnerability to climate change. Combining impacts from habitat loss/fragmentation from forestry and development coupled with fishing harvest (overexploitation) were indicated by experts as a threat to future population resilience of both the Arctic and northern pike populations.

**References**

1 Bradbury C, Roberge MH, Minns CK. Life history characteristics of freshwater fishes occurring in Newfoundland and Labrador, with major emphasis on lake habitat requirements. Minister of Supply and Services Canada; 1999 Apr.

2 Grant, CGJ. and E.M. Lee. Life History Characteristics of Freshwater Fishes Occurring in Newfoundland and Labrador, with Major Emphasis on Riverine Habitat requirements. Can. Manuscr. Rep. Fish. Aquat. Sci. 2004 2672: xii + 262p.

3 Dillon, P.J., B.J. Clark and H.E. Evans. The effects of phosphorus and nitrogen on lake trout (Salvelinus namaycush) production and habitat. 2004 Pp. 119-131 in J.M. Gunn, R.A. Ryder, and R.J. Steedman (eds.). Boreal Shield Watersheds: Lake Trout Ecosystems in a Changing Environment. Lewis/CRC Press, Boca Raton, FL.

4 Guzzo MM, Blanchfield PJ. Climate change alters the quantity and phenology of habitat for lake trout (Salvelinus namaycush) in small Boreal Shield lakes. Canadian Journal of Fisheries and Aquatic Sciences. 2016 Nov 3;74(6):871-84.

5 Whitman A, Cutko A, Walker S, Vickery B, Stockwell S, Houston R. Vulnerability of Habitats and Priority Species. 2014

6 Hare JA, Morrison WE, Nelson MW, Stachura MM, Teeters EJ, Griffis RB, et al. A vulnerability assessment of fish and invertebrates to climate change on the Northeast US Continental Shelf. PloS one. 2016 Feb 3;11(2):e0146756.

7 Sneddon, L.A., and G. Hammerson. Climate Change Vulnerability Assessments of Selected Species in the North Atlantic LCC Region. NatureServe, Arlington, VA. 2014

8 Van Zyll de Jong MC, Lester NP, Korver RM, Norris W, Wicks BL. Managing the exploitation of brook trout, Salvelinus fontinalis (Mitchill), populations in Newfoundland lakes. Management and ecology of lake and reservoir fisheries. 2002:267-83.

9 McCORMICK JH, Hokanson KE, Jones BR. Effects of temperature on growth and survival of young brook trout, Salvelinus fontinalis. Journal of the Fisheries Board of Canada. 1972 Aug 1;29(8):1107-12.

10 Rahel FJ, Olden JD. Assessing the effects of climate change on aquatic invasive species. Conservation biology. 2008 Jun 1;22(3):521-33.

11 Van Zyll de Jong MC, Gibson RJ, Cowx IG. Impacts of stocking and introductions on freshwater fisheries of Newfoundland and Labrador, Canada. Fisheries Management and Ecology. 2004 Jun 1;11(3‐4):183-93.

12 Wade AA, Beechie TJ, Fleishman E, Mantua NJ, Wu H, Kimball JS, et al. Steelhead vulnerability to climate change in the Pacific Northwest. Journal of Applied Ecology. 2013 Oct 1;50(5):1093-104

13 Winfield IJ, James JB, Fletcher JM. Northern pike (Esox lucius) in a warming lake: changes in population size and individual condition in relation to prey abundance. Hydrobiologia. 2008 Apr 1;601(1):29-40.

14 Department of Fisheries and Oceans. Science Advice from a Risk Assessment of Northern Pike (Esox lucius) in British Columbia. DFO Can. Sci. Advis. Sec. Sci. Advis. Rep. 2011 2010/083.
