# Supplementary material for: Assessing the vulnerability of freshwater fishes to climate change in Newfoundland and Labrador
Source: PLoS One. 2018 Dec 3;13(12):e0208182. doi: 10.1371/journal.pone.0208182 (PMC6277096; doi:10.1371/journal.pone.0208182)
Supplement: S2 Appendix — (DOCX) [file pone.0208182.s004.docx]

**S2 Appendix: Drivers of climate change Vulnerability**

**Fig A: Mean experts scores for all climate change vulnerability indicators.** This ranks the factors that drive climate change vulnerability in Newfoundland and Labrador.

**Table A: Mean expert scores for all climate change vulnerability indicators.**

| Species | Exposure to projected temperature | Exposure to projected precipitation | Anthropogenic and non-climatic threat | Habitat specialization | Prey specificity | Physiological/behavioral sensitivity to temperature changes | Physiological/behavioral sensitivity to precipitation changes | Population size/Geographic range | Dependence on environmental cues likely to be disrupted by climate change | Genetic plasticity and evolvability | Dispersive capability | Inherent resilience |
| --- | --- | --- | --- | --- | --- | --- | --- | --- | --- | --- | --- | --- |
| Lake trout | 3.3 | 2.4 | 3.1 | 2.9 | 2.3 | 3.0 | 1.8 | 2.4 | 3.0 | 3.0 | 3.2 | 3.2 |
| Arctic char | 3.9 | 2.6 | 2.8 | 3.1 | 2.3 | 3.5 | 2.1 | 2.6 | 3.0 | 2.9 | 2.8 | 3.3 |
| Atlantic salmon | 3.0 | 2.7 | 3.6 | 2.7 | 2.1 | 2.7 | 3.0 | 2.7 | 3.2 | 2.3 | 1.9 | 3.1 |
| Brook trout | 2.8 | 2.2 | 2.4 | 1.9 | 1.8 | 2.3 | 2.4 | 2.4 | 2.9 | 2.4 | 2.6 | 2.8 |
| Brown trout | 2.1 | 2.5 | 2.1 | 1.8 | 1.8 | 1.7 | 2.1 | 1.9 | 2.6 | 2.2 | 2.3 | 2.8 |
| Northern pike | 1.3 | 1.3 | 1.8 | 1.5 | 2.0 | 1.3 | 1.4 | 1.9 | 2.1 | 2.9 | 3.4 | 3.1 |
| Rainbow trout | 2.1 | 1.9 | 2.0 | 1.4 | 1.7 | 1.8 | 2.0 | 2.1 | 2.3 | 2.3 | 2.4 | 2.6 |
| Total | **3.5** | **2.2** | **2.5** | **2.2** | **2.0** | **2.3** | **2.1** | **2.3** | **2.7** | **2.6** | **2.7** | **3.0** |

**Fig. B: Climate change vulnerability drivers for each freshwater fish.** This indicates the likely drivers of climate change for individual species

**Table B: Expert’s response indicating the non-climatic drivers of the climate change vulnerability across the freshwater fishes.** (i) This corresponds to the number/percentages of responses given by each expert in the assessment that constitute each category. (ii) actual expert’s responses regarding the non-climatic threats to freshwater fishes in Newfoundland and Labrador

(i)

| Species | Habitat loss | Invasiveness/Predation/ competition | Sea mortality | Over exploitation | Disease and pollution | Aquaculture | Total |
| --- | --- | --- | --- | --- | --- | --- | --- |
| Lake trout | 7 (50%) | 1 (7%) | 0 (0%) | 5 (36%) | 1 (7%) | 0 (0%) | 14 |
| Arctic char | 6 (40%) | 1 (7%) | 1 (7%) | 5 (33%) | 1 (7%) | 1 (7%) | 15 |
| Atlantic salmon | 8 (33%) | 4 (17%) | 2 (8%) | 6 (25%) | 2 (8%) | 2 (8%) | 24 |
| Brook trout | 8 (38%) | 4 (19%) | 1 (5%) | 6 (29%) | 2 (10%) | 0 (0%) | 21 |
| Brown trout | 9 (41%) | 3 (14%) | 2 (9%) | 5 (23%) | 2 (9%) | 1 (5%) | 22 |
| Northern pike | 7 (50%) | 1 (7%) | 0 (0%) | 5 (36%) | 1 (7%) | 0 (0%) | 14 |
| Rainbow trout | 5 (31%) | 2 (13%) | 1 (6%) | 4 (25%) | 2 (13%) | 1 (6%) | 16 |

(ii)

|  | **Indicate all possible anthropogenic and non-climate threat to each species and provide an estimate vulnerability level to their cumulative effects.** | | | | | | | |
| --- | --- | --- | --- | --- | --- | --- | --- | --- |
|  | **Atlantic Salmon** | **Rainbow trout** | **Brown trout** | **Brook trout** | **Arctic char** | **Northern Pike** | **Lake trout** |  |
| **Participant A** | instream barriers, forest harvest, fishing harvest | instream barriers, forest harvest, fishing harvest | instream barriers, forest harvest, fishing harvest | instream barriers, forest harvest, fishing harvest | instream barriers, forest harvest, fishing harvest | instream barriers, forest harvest, fishing harvest | instream barriers, forest harvest, fishing harvest |  |
| **Participant B** | - | - | - | - | - | - | - |  |
| **Participant C** | at-sea mortality, invasive species, habitat modification (fragmentation etc.), toxins, harvesting (legal and illegal), aquaculture (genetic, disease and competition) | at-sea mortality, other invasive species, habitat modification (fragmentation etc.), toxins, harvesting (legal and illegal), aquaculture (genetic, disease and competition) | at-sea mortality, other invasive species, habitat modification (fragmentation etc.), toxins, harvesting (legal and illegal), aquaculture (genetic, disease and competition) | at-sea mortality, invasive species, habitat modification (fragmentation etc.), toxins, harvesting (legal and illegal), aquaculture (competition) | at-sea mortality, invasive species, habitat modification (fragmentation etc.), toxins, harvesting (legal and illegal), aquaculture (disease) | invasive species, habitat modification (fragmentation etc.), toxins, harvesting (legal and illegal) | invasive species, habitat modification (fragmentation etc.), toxins, harvesting (legal and illegal) |  |
| **Participant D** | invasives, over exploitation, changing land use, impacts at sea, industrial development | not that relevant, very restricted range | invasives, over exploitation, changing land use, impacts at sea, industrial development | invasives, over exploitation, changing land use, industrial development | over exploitation, less vulnerable (now and in short term) to changing land use and industrial development due to location of populations in unpopulated regions. | changing land use, industrial development | changing land use, industrial development |  |
| **Participant E** | - | - | - | - | - | - | - |  |
| **Participant F** | dams/passage, aquaculture, harvest, habitat destruction | harvest, habitat destruction | dams/passage, harvest, habitat destruction | harvest, habitat destruction | dams/passage, harvest, habitat destruction | harvest, habitat destruction | harvest, habitat destruction |  |
| **Participant G** | Overfishing, loss of habitat, disease, competition with non-native salmonids, predation (seals) | Overfishing, loss of habitat, disease, competition with native salmonids | Overfishing, loss of habitat, disease, competition with native salmonids | Overfishing, loss of habitat, disease, competition with non-native salmonids | Overfishing, | Overfishing | Overfishing |  |
| **Participant H** | habitat degradation, over fishing (incl poaching) | habitat degradation | habitat degradation | habitat degradation / Over fishing in some areas | habitat degradation | habitat degradation / Over fishing in some areas | habitat degradation / Over fishing in some areas |  |
